# Using Adaptive Sensors for Optimised Target Coverage in Wireless Sensor Networks

**DOI:** 10.3390/s22031083

**Published:** 2022-01-30

**Authors:** Junaid Akram, Hafiz Suliman Munawar, Abbas Z. Kouzani, M. A. Pervez Mahmud

**Affiliations:** 1 Department of Computer Science, Superior University, Lahore 54000, Pakistan; junaidakram@superior.edu.pk; 2 School of Built Environment, University of New South Wales, Sydney, NSW 2052, Australia; 3 School of Engineering, Deakin University, Geelong, VIC 3216, Australia; abbas.kouzani@deakin.edu.au (A.Z.K.); m.a.mahmud@deakin.edu.au (M.A.P.M.)

**Keywords:** sensors, wireless sensor network, learning automata, adaptive learning, coverage area, energy efficiency

## Abstract

Innovation in wireless communications and microtechnology has progressed day by day, and this has resulted in the creation of wireless sensor networks. This technology is utilised in a variety of settings, including battlefield surveillance, home security, and healthcare monitoring, among others. However, since tiny batteries with very little power are used, this technology has power and target monitoring issues. With the development of various architectures and algorithms, considerable research has been done to address these problems. The adaptive learning automata algorithm (ALAA) is a scheduling machine learning method that is utilised in this study. It offers a time-saving scheduling method. As a result, each sensor node in the network has been outfitted with learning automata, allowing them to choose their appropriate state at any given moment. The sensor is in one of two states: active or sleep. Several experiments were conducted to get the findings of the suggested method. Different parameters are utilised in this experiment to verify the consistency of the method for scheduling the sensor node so that it can cover all of the targets while using less power. The experimental findings indicate that the proposed method is an effective approach to schedule sensor nodes to monitor all targets while using less electricity. Finally, we have benchmarked our technique against the LADSC scheduling algorithm. All of the experimental data collected thus far demonstrate that the suggested method has justified the problem description and achieved the project’s aim. Thus, while constructing an actual sensor network, our suggested algorithm may be utilised as a useful technique for scheduling sensor nodes.

## 1. Introduction

The rate of advancement in the area of wireless communication is steadily rising. With the increased usage of wireless communication, many devices and applications are developing. Wireless sensor networks are the most popular and growing area of wireless technology (WSNs). These sensors are tiny, low-power, low-cost, multi- functional devices with a short-range communication capability. The network’s sensors main functionalities include detecting, processing, and communicating [1,2,3]. The development of wireless sensor network technology began while military applications for battlefield surveillance were being developed. The development and innovation of new methodologies in wireless sensor networks has expanded the field of application, and it has been used to monitor various fields such as home, disaster prevention, pollution, environmental monitoring, health care, temperature, and so on [4,5,6,7].

The coverage area is another aspect of wireless sensor networks [8,9,10]. This is the region in which a sensor node observes and tracks the activities of the chosen target. Each target should also be constantly monitored by at least one of the sensor nodes to ensure network operation continuity. While doing so, effort has been taken to ensure that energy is used efficiently. For this, the network’s nodes have been maintained in two states: active and sleep. Nodes in the sleep state are considered OFF (use no energy), while nodes in the active mode monitor their surroundings or targets. The network’s lifespan has been extended by programming each sensor’s activity in active and sleep modes [11,12,13].

A lot of study was done on coverage in order to develop an energy-efficient wireless sensor network. Various scheduling methods are developed for this aim to plan the activity of the sensor nodes [14,15,16,17]. Learning automata is one of the planning techniques for an energy efficient wireless sensor network. This technique enables the sensor switch to detect its current condition and choose the appropriate state to extend battery life [18,19,20,21].

At this point, it is assumed that all of the sensor nodes are operational and that they are monitoring at least one target. As a result, the sensor nodes are planned with active and sleep modes to ensure that the network is as efficient as possible and that battery consumption is kept to a minimum. The sensor node is supplied with a sleep mode of state if it is not monitoring the target, and an active mode of state if it is monitoring the target. The scheduling method based on adaptive learning has been developed for this idea. During network operation, each node is endowed with a learning automaton, which allows it to choose one of two states: active or sleep. A node does not consume energy while it is in sleep mode, but it does when it is in active mode.

### Problem Statement

*Using adaptive learning, how can a wireless sensor network achieve energy-efficient target coverage?*

Consider a collection of M targets, indicated by M = M1, M2,…, Mm, that are monitored by a set of N sensor nodes, denoted by N = N1, N2,…, Nn, and that these two sets are deployed in a XxX area, with each sensor node covering all targets. Each experiment has a defined sensing range “R” for all sensor nodes. There are also more sensor nodes than targets, according to the assumption. This is because it is necessary to keep track of targets in sensor networks. This helps to save energy and extend the life of the network. The sensor nodes scheduling algorithm has been presented to schedule. This will provide you the status of the sensor nodes. A sensor node can be in one of two states: active or sleeping. The sensor node’s status is active if the target is within its range. The target is in a sleep state if it is beyond the sensor’s range. The sensor consumes some energy when it is active, but it accomplishes nothing when it is asleep. The sensor node is said to cover a target point Mj within the range 1 ≤ j ≤ M. 1 ≤ I ≤ N, if it is inside the range of one of the sensor nodes.

The project use the following notations:

- N is the number of sensors;
- M denotes the number of targets;
- X sensor and target deployment area;
- R, the sensor node’s sensing range;
- Γ, the initial active time duration of both sensors and targets;
- Ni is the ith sensor node such that 1 ≤ i ≤ N;
- Mj, the jth target in 1 ≤ j ≤ M.

The primary goal is to arrange sensor node activity in order to maximise network lifespan.

A collection of M targets M = M1, M2,…, Mm and N sensor nodes N = N1, N2,…, Nn are taken in a X region for sensor deployment. Sensors are installed redundantly in such a way that each sensor may monitor at least one target to obtain a maximum lifespan.

The adaptive learning automata technique was used to schedule the sensors. This technique will assist sensor nodes in selecting their state based on their probability vector without interfering with the network’s functioning. This learning automata approach assists sensor nodes in selecting their proper state based on the network’s operational requirements. The initial Euclidean distance between the sensor node and the targets was determined to determine target coverage. The sensor compares euclidean distance to sensor detecting range to see if the target is within their range. The sensor is monitoring the target if the computed Euclidean distance is smaller than the sensing radius “R.” Sensor switches to the active state at this point. Otherwise, it will remain dormant and in a sleep condition. The sensor consumes some energy when it is active and none when it is in the sleep state.

The suggested technique would choose the best active sensor nodes from all active sensor nodes that cover the most targets after many iterations. As a result, the active sensor count is lowered. This means that utilising fewer active sensors uses less energy, resulting in total network energy saving and increased longevity.

The following issues will be addressed by this research work:

- What is the best way of calculating the number of active sensors?
- How should the sensor node be scheduled for maximum energy conservation in a wireless sensor network?

## 2. Related Work

There is a description of how to handle the target coverage issue in [22,23,24,25,26]. As a result, there is a need to manage goals in order to get the greatest lifetime. The paper utilised a probabilistic coverage model to achieve this goal.

Sensors may be split into cover sets to provide sufficient target coverage. This cover set includes a time period for monitoring targets. This solution, however, has been shown to be NP-complete. As a result, authors in [27,28,29] have suggested a centralised heuristic method to achieve coverage. Cover sets are generated by this method, which aid in the monitoring of all targets.

There are a few techniques in the future that focus on the bio inspired algorithms to solve the issue of energy conservation in sensor networks. Rango et al. [30] suggest a technique for exploring an unfamiliar region based on ants’ behaviour, as well as an unique swarm-based protocol for recruiting and coordinating robots for jointly disarming mines. The usefulness of the proposed ant-based task robot coordination (ATRC) with simply the exploration task and combined exploration and recruitment procedures is demonstrated through simulation experiments. The robots’ recruitment time and the overall area exploring time have both been regarded as reduction targets. Palmieri et al. [31] presented a restricted bi-objective optimisation problem in which mobile robots must undertake two distinct tasks of exploration while also cooperating and coordinating to disarm the dangerous targets. These are diametrically opposite aims, with one valued only at the expense of the other. As a result, a good trade-off must be made. This problem is solved using a nature-inspired method and an analytical mathematical model that considers a single equivalent weighted goal function. The suggested coordination model’s findings are shown in a two-dimensional terrain simulation to analyse the applicability of the system workable solution. The authors investigated the approach’s performance and the impact of the goal function’s weights in static and dynamic situations. In this case, the robots may fail due to a strict energy budget or dangerous circumstances. Palmieri et. al. [32] explores the use of several bio-inspired metaheuristics for the coordination of a swarm of mobile robots that must explore an unfamiliar environment in order to rescue certain scattered targets. This issue is defined by first constructing an optimisation model, then examining two subproblems: exploration and recruitment. To begin, robots use a modified form of ant colony optimisation to progressively investigate the area. When a robot recognises a target, a recruitment mechanism is activated to enlist additional robots to help with the disarming job. Authors developed and compared three techniques based on three distinct bio-inspired algorithms for this goal (artificial bee algorithm, particle swarm optimization and firefly algorithm). Simulation findings show that, in most cases, the firefly-based technique outperforms the competition and saves energy, especially in complicated settings. Our Work differs from the above-mentioned studies as we are intending to use the least amount of sensors to cover a target. This, in return, minimises the overall energy consumption of the whole network.

P2P networks are built on data retrieval methods, and this study tackles the challenge of effectively searchingf for files in unstructured P2P systems. We present a completely distributed and bandwidth-efficient improved adaptive probabilistic search (IAPS) method. In order to find file container nodes with a high likelihood of success, IAPS employs ant-colony optimisation and takes file kinds into account. We studied the performance of IAPS using extensive simulations and compared it to the random walk and adaptive probabilistic search algorithms. IAPS achieves high success rates, high response rates, and considerable message reduction, according to our testing data [33].

In [34,35,36], you may find the solution to the issue of how to manage the schedule of a big number of sensor nodes installed. The maximum lifetime coverage issue is presented in this article as a big sensor deployment. As a result, the authors offer a solution to the aforementioned issue by using a polynomial-time approximation method.

The network’s lifespan is determined by the channel used to carry data from the environment to the sink. The suggested technique aims to find the most cost-effective data transmission path. This study examines efficient routing approaches for maintaining k-coverage in a sensor network, and then provides a strategy for preserving k-coverage and data dependability while allowing for logical fault tolerance. Network nodes are supposed to be aware of how much energy they and their neighbours have left. The two types of sensors are coverage and communicative nodes, with some being classified as clustering and dynamic nodes in the process. The proposed approach, according to simulation results, uses energy more effectively [37].

Because the WSN node has a limited power backup, this is a highly important problem to address. It is proposed in the paper [38,39] that wireless sensor networks (WSNs) can benefit from computational intelligence techniques such as multi-objective particle swarm optimization (MOPSO), with the overall goal of concurrently minimising localization time, energy consumption during localization, and maximising the number of nodes that are fully localised. Using Dijkstra’s algorithm, refs [40,41,42] construct an improved version 15 July 2021 submitted to Mathematics 3 of 19 versions of the low-energy adaptive clustering hierarchy (LEACH) protocol in a cloud environment. This protocol optimises the power consumption or energy usage based on shortest route selection, and it is referred to as LEACH-DA. Refs [43,44] utilise round robin, throttled, active virtual machine, particle swarm optimization, ant colony optimization and odds algorithm for efficiency.

The k-coverage is described as the user-specified region of sensor coverage where the sensor may cover its objectives, according to [45]. In the article, two methods were proposed for this purpose. The first is the PCL-greedy-selection (GS) method, which is used to deal with sensors that have a defined sensing range and are part of disjoint subsets. The PCL-greedy selection-adjustable (GSA) method comes next, which works with sensors that belong to non-disjoint subsets and sensors that have adjustable sensing range capabilities. The study found that the GSA method is a superior approach for solving the k-coverage issue in wireless sensor networks after comparing the two techniques.

To ensure the wireless sensor network is durable, the actions associated with the cover sets must be scheduled which are sensor node groups that cover a certain number of targets. As a result, in order to acquire these cover sets, sensor nodes must be placed in such a way that target coverage is attained. To determine the network lifespan, the authors of [46,47,48] used a heuristic and an artificial bee colony method. As a result, the authors suggested two scheduling methods for sensor deployment: heuristic and ant colony. The solution to the cover set issue, as stated in the preceding article, is explained in [49,50,51,52]. The authors described the learning automata algorithm in this paper. This method is used to plan the nodes’ sensor activity in order to optimise the network lifespan.

Target coverage, as well as the issue of data gathering in WSNs, are addressed in [53,54]. This information gathering aids in the transfer of sensed data from nodes to sinks. The issues described previously are examined using polynomial-time approximation and polynomial-time constant approximation techniques. Experiments are carried out using sensors that have the same sensing and transmission radius. To exchange information, the sensor interacts with its neighboring node. This sensor necessitates the usage of some energy. However, high data rate transmission requires a significant quantity of energy. As a result, this sensor network problem should be taken into account throughout the course of the network’s lifespan. For this, [55,56,57,58] proposes an energy-efficient pattern for high data rate transmission in wireless sensor networks without compromising sensing coverage rate.

If there is a need to deploy sensor nodes in a wide region with a significant number of sensor nodes, such as for environmental risk monitoring. To improve throughput, a suitable monitoring algorithm is required. In [59,60,61,62], a physics-based heuristic method is presented. This method aids in the effective placement of scalable sensor nodes in wireless sensor networks to determine coverage of fixed objectives. The idea of virtual sensors was proposed in this paper. This is used to move and merge sensors in order to obtain the smallest number of target-covering sensors possible.

In [63,64,65], the author discusses how to choose suitable coverage methods for improved wireless sensor device performance in the industry. This article presented four methods for solving the target coverage issue. The average energy usage, coverage time, network lifespan, and other characteristics of these four algorithms were examined. The results are used to choose the best methodology for improving industrial WSN connection and coverage.

The key goal and essential sensor aspects are highlighted in [66] for optimising the network lifespan of the wireless sensor network. This important target is covered by the fewest number of sensors, and this sensor is referred to as critical sensors. The author developed a heuristic for choosing a small number of essential sensors to extend the life of sensor networks. Furthermore, by covering all targets, this sensor will cover important targets for a longer period of time.

The novelty in our approach, looking at the state-of-the-art, is that the sensors will be scheduled using the adaptive learning automata approach. This method will let sensor nodes choose their state depending on their probability vector without interfering with the network’s operation. This learning automata technique will aid sensor nodes in determining the best state for them depending on the operational needs of the network. To assess target coverage, the initial Euclidean distance between the sensor node and the targets will be calculated. To determine if the target is inside the sensor’s detection range, the sensor compares Euclidean distance to the sensor’s detecting range. If the calculated Euclidean distance is less than the detecting radius “R,” the sensor is monitoring the target. At this point, the sensor enters the active state. Otherwise, it will remain inert and in sleep. When the sensor is operating, it needs some energy and when it is asleep, it uses none.

## 3. System Model

This section goes through the environment setup and algorithm utilised in this project in greater depth. This section will also discuss how design is applied and formulated in accordance with the plan. The design is more easily conveyed when figures, tables, and algorithms are used.

The design step is broken down into smaller chunks in this section. The plans indicated in the approach section are explained in this section. This section describes the circumstances that were employed to execute the project. There is a description of what standards and rules to follow when designing our project in accordance with the plan’s flow.

### 3.1. Sensor Deployment

This provides a description of how the project’s sensors are distributed and the tactics used to do so. Uniform random, square grid, and tri-hexagon tiling are some of the sensor node deployment models. The random model is employed in his project uniform. As a result, the sensors are placed in a rectangle region at random, each having the same sensing range. The number of sensors is always assumed to be more than the number of deployed targets in this case. All sensors have an equal chance of covering the targets in the initial condition. The suggested learning automata technique aids the sensor node in learning its state automatically in each iteration as the probability value changes based on its state. The sensor network’s coverage is calculated using the distance formula. The distance between the sensor node and the network’s target is calculated using this formula. The sensor is covering the target if this obtained value is less than or equal to the sensor sensing range. Otherwise, the target is beyond the sensor node’s range. The Algorithm 1 represents the sensor deployment working:
**Algorithm 1: Sensor Deployment.** Grid size = 600; Range sensor = 100; Number of sensors = 20; Number of Targets = 10; x sensor = [0]* Nsensors y Number of = [0]* Nsensors; x Targets = [0]* M Targets; y Targets = [0]* M Targets; for i→ Number of sensors do      Initialize x_sensor and y_sensor with random number;     plot the points with text;     Scatter plot the sensors;     assign circular coverage of Sensors; end

The variable N Sensors in the above algorithm represents the number of sensors that will be deployed. For example, in this script, N Sensors is set to 20 with a sensing range of 100 and a network grid size of 600 m. The sensor’s value fluctuates depending on the experiment’s requirements. The x sensor [i] and y sensor  [i] variables determine the sensor’s position. This is the sensor’s position within the x- and y-axes.

### 3.2. Target Deployment

In this project, the target is used as a sensor at random. The position of the targets is random. The number of targets placed is always smaller than the number of sensors deployed. As a result of the assumption, there should be at least one sensor that covers the target. The Algorithm 2 for target deployment is given as:
**Algorithm 2: Target Deployment.** Grid size = 600; Range sensor = 100; Number of sensors = 30; Number of Targets = 25; x sensor = [0]* Nsensors y Number of = [0]* Nsensors x Targets = [0]* M Targets; y Targets = [0]* M Targets; for i→ Number of targets do      Initialize x_sensor and y_sensor with random Number;    plot the points with text;    Scatter plot the targets;end

### 3.3. Proposed Approach

**Figure 1:**
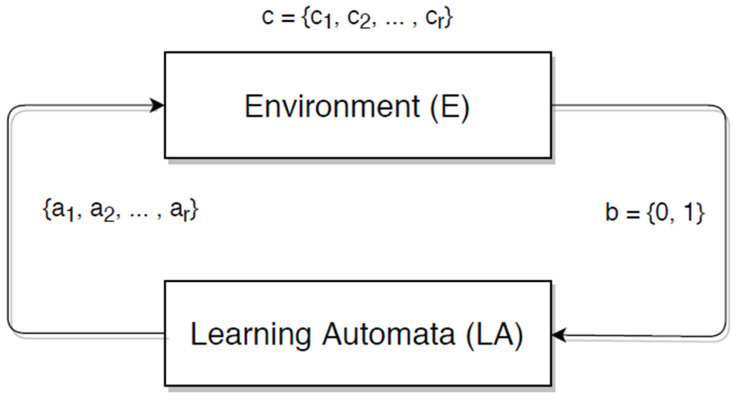
Projection of learning automata and random environment association [43].

Learning automata are a machine learning paradigm. This is an abstract model that chooses the best action from a limited number of options and executes it in a complex environment. The environment then analyses the chosen action and reacts to the automaton using the reinforcement signal environment. To choose the next action, the automaton must first update its internal state from the previous action and then receive a signal. Thus, by following a set of rules, an automaton can discover the optimal output solution and interact with the environment in order to make the best choice for appropriate action, as shown in Figure 1.

The environment is composed of three models. They are given as follows:

- P-model: In this paradigm, b can only accept binary values of 0 or 1.
- Q-model: This model’s environment allows finite output sets to have numerous components that take values in the [0, 1] range.
- S-model: The environment’s output in this model is a continuous random variable that takes values in the range [0, 1].

In addition, there are two types of learning automata: fixed and variable structure stochastic models.

A learning algorithm may be stated in formal terms as follows:

(1) P(n+1)=T[p(n),a(n), b(n)]

The actions selected are p(n) and a(n). The moment at which an action is carried out is called n. The action is based on the probability vector of the action p. The action probability vector p is updated using the repeating equation below, which is a linear learning method. Let a1 (n) represent the chosen action at n.

(2) $$p_{i\;}\left(n+1\right)=p_{i\;}\left(n\right)+a\left[\;1p_{i\;}\left(n\right)\right]$$

(3) $$p_{j\;}\left(n+1\right)=\left(1-a\right)\;p_{j}\left(n\right)\forall j,\;j\neq i$$

when the environment favours a certain behaviour (i.e., b(n)=0) and

(4) $$p_{i\;}\left(n+1\right)=\left(1-b\right)p_{j\;}\left(n\right)$$

(5) $$p_{j\;}\left(n+1\right)=\frac{b\;}{r-1}+\left(1-b\right)p_{j}\left(n\right)\forall j,\;j\neq i$$

when the environment punishes a certain behaviour (e.g., b(n)=1).

The punishment and reward are represented by b and a, respectively, and the number of reductions and increases in action probability is calculated. The number of actions performed is represented by r. $p_{i}\left(n\right)$ and $p_{j}\left(n\right)$ reflect the probability of the acts $a_{i}$ and $b_{i}$, respectively [67,68]. Linear reward–inaction (LR I) algorithm for b<<a, linear reward epsilon penalty $\left(L_{R}-\epsilon_{P}\right)$ method for b=0, and linear reward–penalty $\left(L_{R}-P\right)$ algorithm for b=0. The Algorithm 3 for initial phase is given as:
**Algorithm 3: Initial Phase.****Input:** Given, N number of sensors, M number of targets and R sensor sensing range, a end e learning automata parameter **Result:** Number of active sensors that cover the targets Initialize the Networks parameters: Calculate the distance between sensor and target; Determine the coverage and covered targets in the network; **while**
*All targets not covered* do **If** (*Range of sensor* ≥ *distance between sensor and target*) **then**    Set sensor mode to active;   Add them to list of active sensors by using learning algorithm;   **else**    Set sensor mode to sleep by learning algorithm;      **End** Iteration = iteration + 1 **end**

The algorithm aids in the selection of the most effective active sensors capable of monitoring the greatest number of targets. The first phase begins with the sensor node’s information being disseminated to its neighbours through a message. It comes to an end when a neighbour responds with information on the watched targets. The learning phase then begins by utilising learning automata to choose a node’s state and concludes with the proper probability vector value for the whole process. The target monitoring phase is the last step, which begins with learning automata to choose the optimal action of sensors and concludes with a sensor that operates in accordance with that action.

There are three phases to this algorithm.

### 3.4. Initial Phase

During this phase, all of the network’s sensors are given learning automata $LA_{i}$. This aids the sensor node in determining whether it should be active or sleep. The chance of choosing the state is set at 0.5 in the early stages. The project’s sensor nodes are deemed self-contained. That is, they initiate contact and send messages to their neighbour node on their own, providing their ID, location, and list of covered targets. As a result, they know which state to choose in any circumstance. The learning and target monitoring phases follow this one.

### 3.5. Learning Phase

Each sensor node is provided with learning automata at this step. The node is chosen at random at first. Select the state of the node using purposed learning automata. Then it sent the message packet to its neighbours, containing all of its information. All nearby nodes respond to their status by listening to this message. The sender then takes the following steps:

○ If the sender node’s Learning Automata $LA_{i}$ state is active and the Replying Neighbor node is also active, the sender node goes to sleep by lowering its probability vector value for covering the target.
○ If the sender node’s Learning Automata $LA_{i}$ state is active and the Replying Neighbour node is likewise asleep, the sender node will be active and will cover the network’s goal. As a result, the probability vector of covering the target will rise. The Algorithm 4 for learning phase is given as:

**Algorithm 4: Learning Phase.****Input**: Number of active sensors that cover the targets **for** N number of sensor in network **do**    for Each action of sensor node **do**        initial probability = 0.5;   **end** **end**for i = 0 to iteration **do**   **for** Every Node in Network do      Node = choose random action;     Send action packet to neighbour nodes;     Receive action packet from all neighbour nodes;     **If** Node action is sleep and neighbour node could cover the target **then**         set the action mode of sender node to Sleep;         Decrease the probability vector to cover;         he target by using learning automata;     **else**
         Increase the probability of sender node to cover the target          **end**     **if** Node action is Active and neighbour node could not cover the          Target then Then Set node mode to Active state;         Increase the probability vector of;         Learning automata to cover the targets;     **else**
         Go to sleep mode using learning automata     **end**
   **end****end**

This phase will continue until all of the network’s targets have been covered. The likelihood of an active state sensor node increases with each cycle of operation.

### 3.6. Target Monitoring Phase

In this phase, the sensor nodes select its state for the whole operation as an active or sleep. The Algorithm 5 for Target Monitoring Phase is given as:
**Algorithm 5: Target Monitoring Phase.****Input:** Number of active sensors that cover the targets for N number of sensors in network **do**  Select Best action (); **If** Best action () = Active **then**     Node state = Active;     Current Coverage set = Current Coverage U Node;  **else**     Node State = Sleep;  **end****end** Monitor the target till end of target monitoring phase;

## 4. Result and Discussion

This section goes over the many implementation phases, the findings that were acquired, the problems that were encountered, and the various error sources that might have impacted the experiment.

The suggested approach, known as the Adaptive Learning Automata Algorithm (ALAA), is also assessed here. The evaluation is carried out by running a network simulation on a computer with sensors and targets placed at random in a 600 m × 600 m region.

The following parameters are utilised in all of the experiments:

- N is the number of sensors that are placed at random. Its value ranges from ten to eighty.
- M is the number of targets that are deployed at random. Its value spans from 4 to 60.
- R is the sensor’s sensing range. Its value varies between 50 and 600 metres.
- To obtain a result, the experiment is repeated 1000 times.
- The script makes use of a random seed. This allows you to use the same setup for all of your tests.
- Parameter for learning:
  Lambda (λ) has a range of values between 0.0001 and 0.4.
  Epsilon(e) has a constant value of 0.01 throughout the experiment.

All the experiments have been performed on google colab notebooks using Python programming language. The algorithm is also coded in Python on a colab notebook. Only the parameters that need to be examined are modified for the experiment, while the others stay constant. The following experiments have been performed to check the validity of the proposed technique:

1. A minimum number of active sensors covering all targets is discovered by adjusting the sensing range of sensors.
2. To attain the smallest number of active sensors, experiment with altering the number of sensors while maintaining a set sensing range.
3. Determine the effect of target density on discovering the smallest number of active sensors.
4. Examine the suggested learning ALAA’s efficiency. This is accomplished by altering the proposed algorithm’s parameter. The learning automata algorithm is changed in this case by keeping e constant during the whole procedure.
5. Create a 2 n binary combination of sensors to test the validity of the experimental results, where n is the number of sensors utilised in the experiment. This is referred to as a brute force approach. It pulls just the active sensor combinations that cover all targets from these combinations. This is used to determine whether the combination obtained is accurate. This brute force approach generates a list of all conceivable active sensor combinations. This list is compared to a previously obtained list in order to verify the proposed algorithm’s output.
6. Analyse the impact of sensor and target density on learning automata operation.
7. Compare the effectiveness of the suggested learning automata to the work of others (LADSC scheduling algorithm).

**Figure 2:**
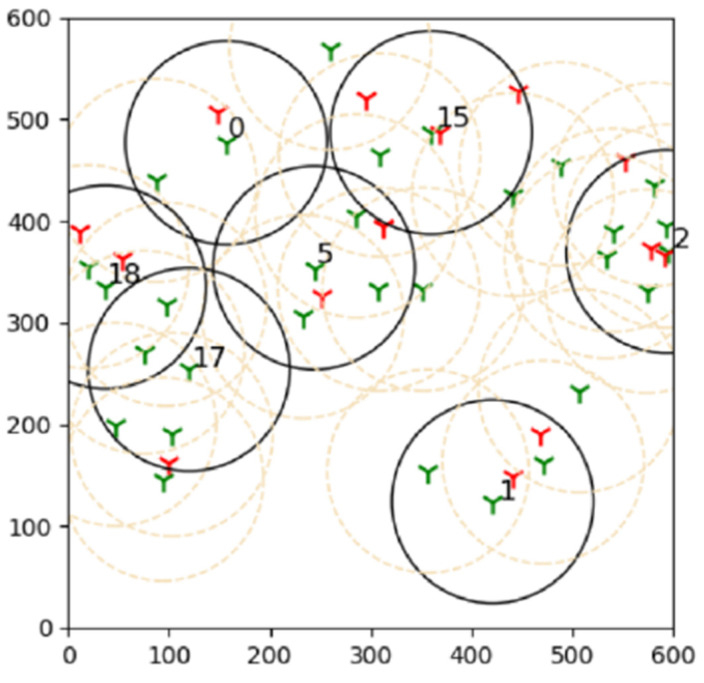
One of the final simulation results of the experiment where 7 active sensors are able to cover all 15 targets. The sensing range is 200 m.

An experiment is conducted with the main objective to see how sensing range and sensor density affect the performance of a large network. A network with a high number of sensors and objectives is referred to as an extensive system. The experiment begins by changing the sensor range between 150 and 600 m with 70 sensors and 50 targets. (Figure 2).

**Figure 3:**
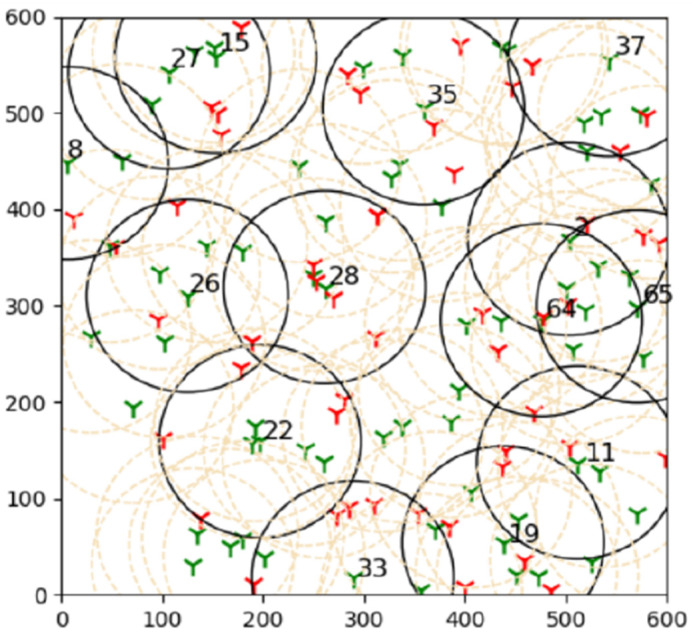
The influence of sensor density and sensing range on getting the average minimum active sensors in a large network is shown in this simulation.

The next test is to increase the density of sensors in order to evaluate the algorithm’s performance and get the smallest number of active sensors. (Figure 3).

**Figure 4:**
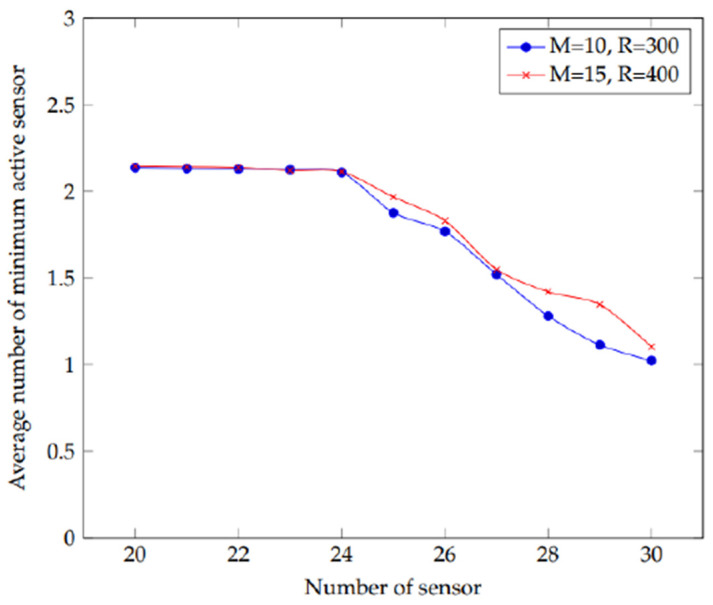
Plot depicting the effect of increasing the number of sensors on the average number of active sensors in the experiment.

**Figure 5:**
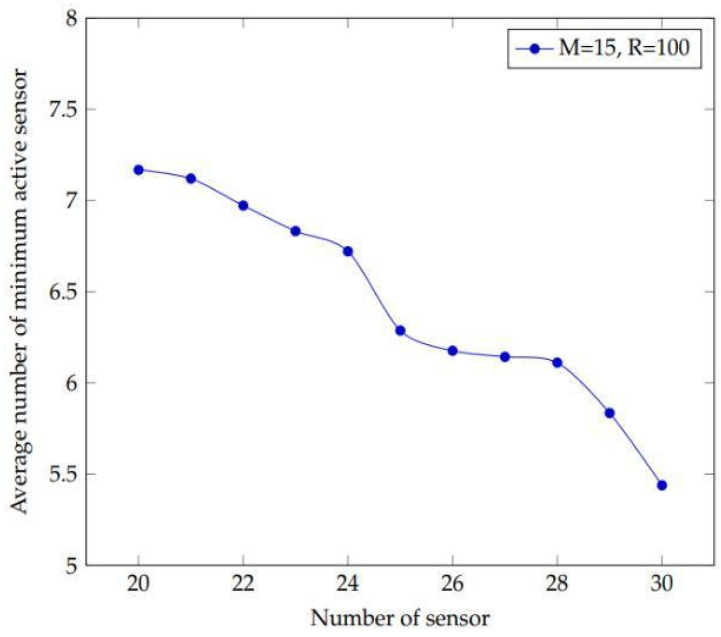
Plot depicting the effect of increasing the number of sensors in the experiment by using 15 targets and a minimal sensing range of 100 m to achieve the average minimum active sensors.

The experiment is carried out to look into the effect of a certain number of sensors on the network’s active sensors. This sensor number is chosen from a range of 20 to 30 with a one-step increment. All the distances represented in the plots and in metres. Figure 4 shows the results of 10 and 15 targets with sensing ranges of 300 and 400 m, respectively. Figure 5 shows 15 targets having a sensor range of 100 m. Figure 4 depicts the collected data, which demonstrate that as the number of sensors increases, the number of active sensors decreases. Furthermore, there is little influence on the network when the number of targets and sensing range grows. As a result, both curves are equidistant. This indicates that as the number of sensors grows, the demand for active sensors decreases. As a result, the network’s lifetime can be extended.

The results of using a small sensing range are represented in Figure 5. It illustrates the requirement for extra sensors to cover the targets when the sensing range is limited. However, when the detecting range increases, the number of active sensors needed to cover the target decreases, displaying the same pattern of findings as Figure 4. As a result of this experiment, one can see how the number of active sensors decreases as the number of sensors increases. Furthermore, irrespective if a big or small sensing range is used, the results follow the same pattern.

**Figure 6:**
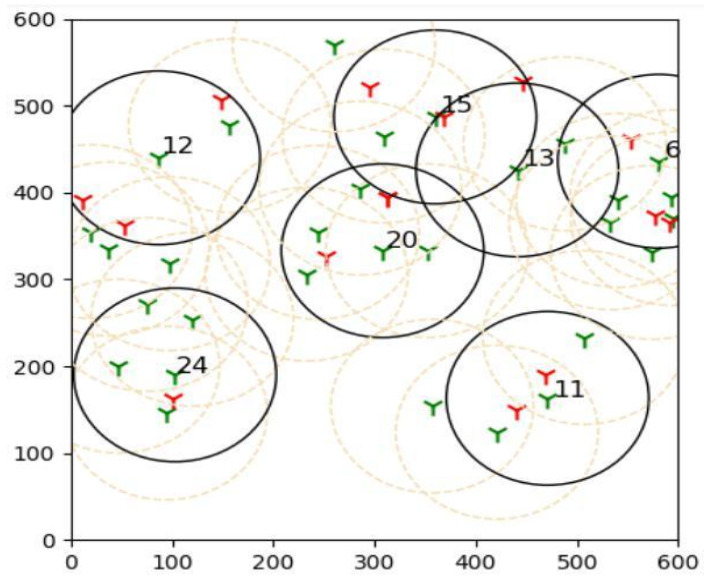
The experiment’s simulation result demonstrates the influence of sensor density in a network.

As a result, the number of sensors that must be active to monitor the target is reduced. All other sensors that are not in an active condition can be put to sleep to preserve battery life. They can also be utilised in the following phase of testing. Based on the foregoing findings, it can be concluded that as the detecting range of a sensor increases, the number of active sensors in a vast network decrease as shown in Figure 6.

**Figure 7:**
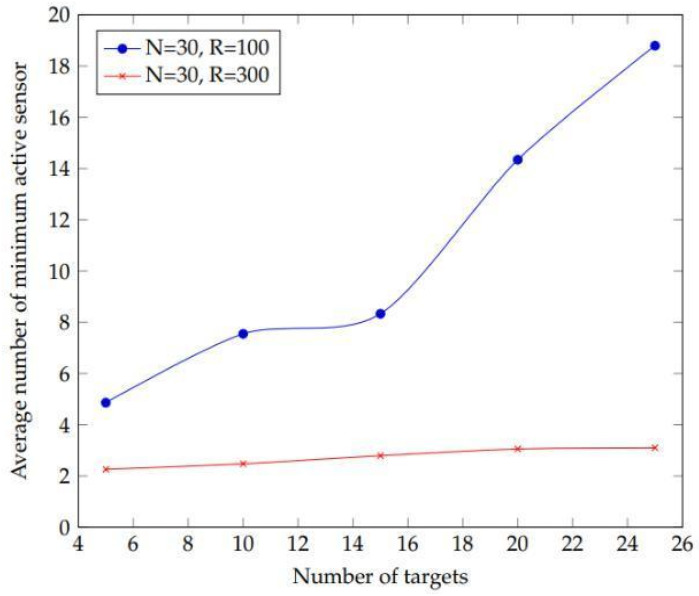
Plot depicting the effect of increasing the number of targets on achieving the experiment’s average minimum active sensors.

An experiment conducted to see how target density affects the average number of active sensors in a network. The goal is chosen between 4 and 26 with 30 sensors and a sensing range of 100 m in order to do this. Figure 7 depicts the outcome of the experiment. This finding shows that the number of target sensors and the average minimum active sensors have an inverse relationship. This means that as the number of targets grows, so does the number of active sensors required to cover them. However, when the number of targets grows, there is an influence of sensing range.

**Figure 8:**
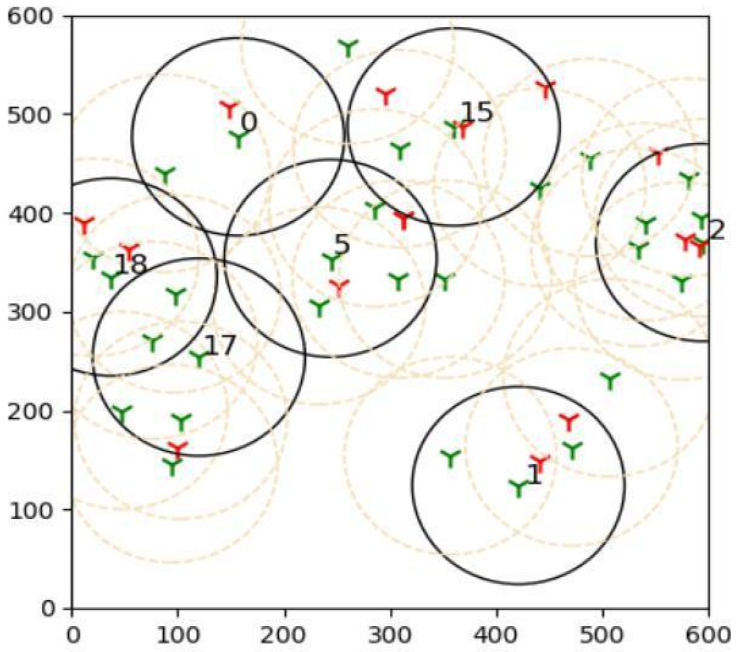
The effect of target density on achieving average minimum active sensors was simulated.

This result suggests that our suggested method will have a high scheduling capacity. To put it another way, the suggested method is aided by the lowering value of the learning parameter value in scheduling the sensor nodes’ activity more effectively. As a result, the network lifespan is maximised by using the fewest possible sensors. It also demonstrates that, in order to get the optimum results, the value of the learning parameter should be varied for a big network with a large number of sensors (Figure 8).

**Figure 9:**
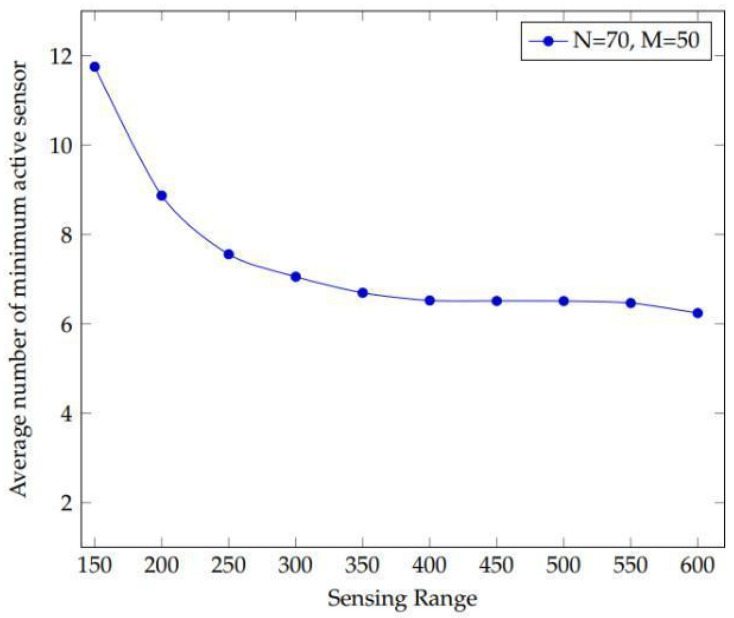
A large network of 70 sensors and 50 targets with a sensing range ranging from 150 m to 600 m.

Figure 9 shows that as the sensing range increases, there is a consistent declination in the plot. This has the effect of extending the sensor’s detecting range in a big network. This is due to the fact that when the detecting range grows, the sensor’s covering area expands. The larger the coverage area, the more likely it is that more targets will fall inside this range. As a result, it can keep track of the number of targets.

**Figure 10:**
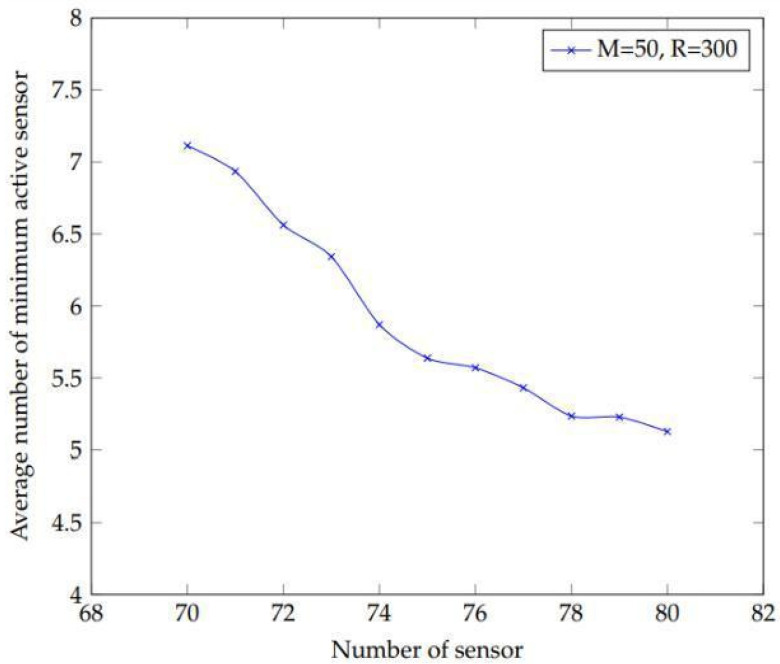
The outcome of a big network with 50 targets and a sensing range of 300 m, with the number of sensors increasing from 70 to 80.

Figure 10 depicts when a vast network of sensors is deployed then there is a better chance of obtaining fewer active sensors to keep an eye on the targets. To get these results, reduce the value of the probability parameter as the number of sensors grows. Another experiment has been conducted with the primary objective to investigate the link between the learning parameter rate and the number of active sensors. Two parameters are employed in the suggested method. Lambda (λ) and epsilon (∈) are two examples. To obtain the findings, the lambda “l” parameter of learning automata is changed.

**Figure 11:**
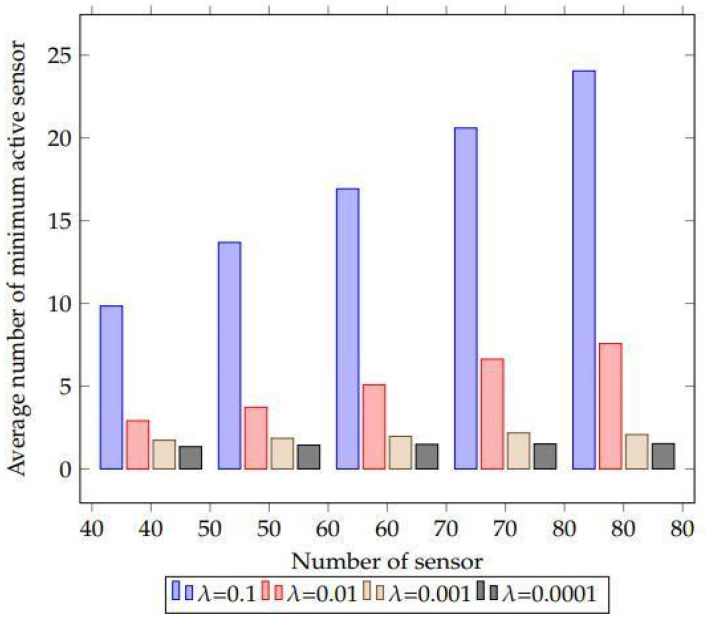
The effect of reducing the value of learning parameter values is shown as a bar plot.

The linear curve of results is obtained using the brute force approach (Figure 11). However, the suggested learning algorithm’s outputs the first slope to some extent before showing linear behaviour. This demonstrates that brute force produces better results than the suggested technique. As a result, it aids in determining the efficacy of our suggested algorithm in achieving our aim.

**Figure 12:**
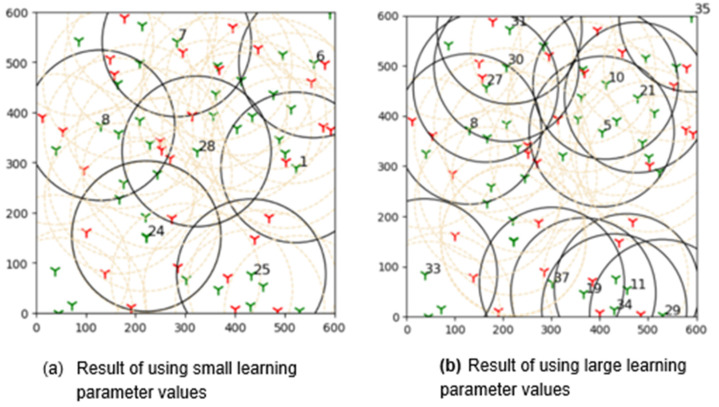
The simulation results of the experiment to evaluate the influence of the proposed learning automaton algorithm’s learning parameter.

Sensors ranging from 40 to 80 are used with 30 targets in this experiment. Each sensor has a detecting range of 400 m. The experiment is then formulated by using several values of the learning parameter lambda “λ”. The values of lambda are “ λ = 0.1”, “ λ = 0.01”, “*λ* = 0.001”, and “*λ* = 0.0001” in this case. Figure 12 summarises the findings. As a result of this finding, one may deduce that the demand for an average number of minimum active sensors decreases as the value of the learning parameter decreases. However, when the value of the learning parameter is reduced, the number of iterations increases, resulting in more exact outputs. As a result of this phenomena, algorithm execution time and memory consumption both rise. Another rationale is that as the number of sensors grows, the learning parameter value should decrease in order to achieve the optimum outcomes.

According to the findings, when the number of sensors in the deployed environment grows, a modest value of the learning parameter must be used to get satisfactory outcomes. This allows for the employment of fewer active sensors to keep track of all the targets. As a consequence of this finding, the suggested method offers optimal scheduling to enhance the network lifetime with an increase in the sensor network with a small amount of learning parameters.

**Figure 13:**
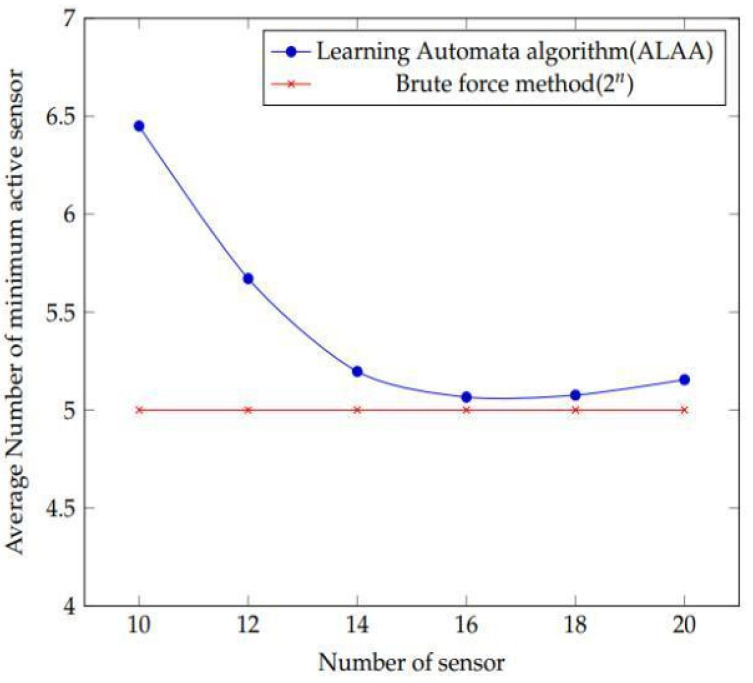
A graph of the results of the 2 n technique and the brute force approach is shown in this diagram. The figure shows the number of active sensors for each algorithm, along with the targets that they cover. Using these methods, the influence of sensor density on obtaining an average number of minimum active sensors is assessed for a sensor number increasing from 10 to 24 with a sensing range of 100 m. Based on the obtained findings, it can be concluded that our suggested scheduling algorithm outperforms the LADSC method in terms of getting a lower number of active sensors to monitor all targets. Even while the LADSC algorithm appears to use fewer active sensors in some cases, it is unable to cover the entire target. As a result, it is inefficient in comparison to our method. This means that our method consumes less energy than the LADSC algorithm, allowing us to extend the lifetime of a sensor network. Finally, the best outcomes possible are acquired.

These methods are compared first on the basis of sensor density, and subsequently on the basis of sensor range. The algorithms are rated based on their ability to use the fewest number of best active sensors while still covering the targets. For the first comparison, 9 targets with sensors ranging from 9 to 25 with a detecting range of 100 m are used. In the second comparison, 15 targets are used, the sensor’s detecting range is increased from 50 to 100 metres, and there are 20 sensors in total, as shown in Figure 13.

The purpose of this experiment is to see how accurate our suggested learning automaton algorithm’s findings are. The experimental setup consists of nine targets and a number of sensors ranging from ten to twenty. All sensors have the same detecting range, which is set at 100 m. The experiment then begins by utilising the suggested learning automata method to determine the number of average minimum active sensors. Then, using the same configuration, brute force testing is carried out. The deployed number of sensors is multiplied by 2 n in this brute force technique. The number of sensors utilised in the experiment is denoted by “n”. Only the best active sensors are produced as a result of this combination. The best outcomes are found by observing and comparing the results of both approaches.

**Figure 14:**
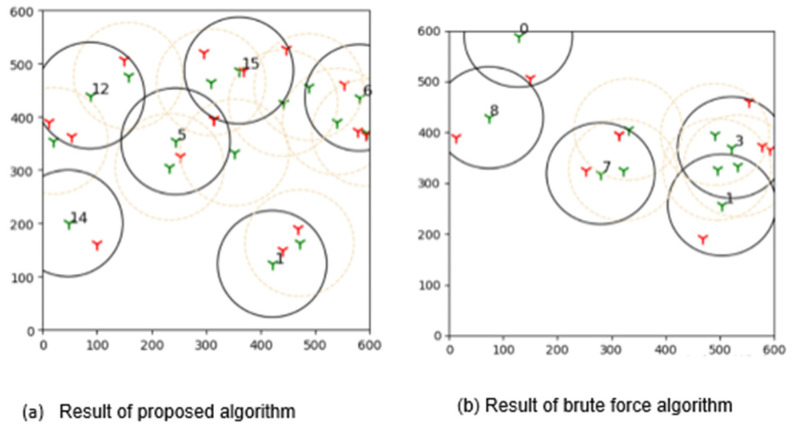
Simulation result obtained after formulation of the experiment using the proposed learning automata algorithm and brute force method.

Figure 14 depicts the outcomes of the experiment. As can be seen, the results achieved using the brute force technique (2 n) and the suggested learning automata algorithm (ALAA) method are similar. 

**Figure 15:**
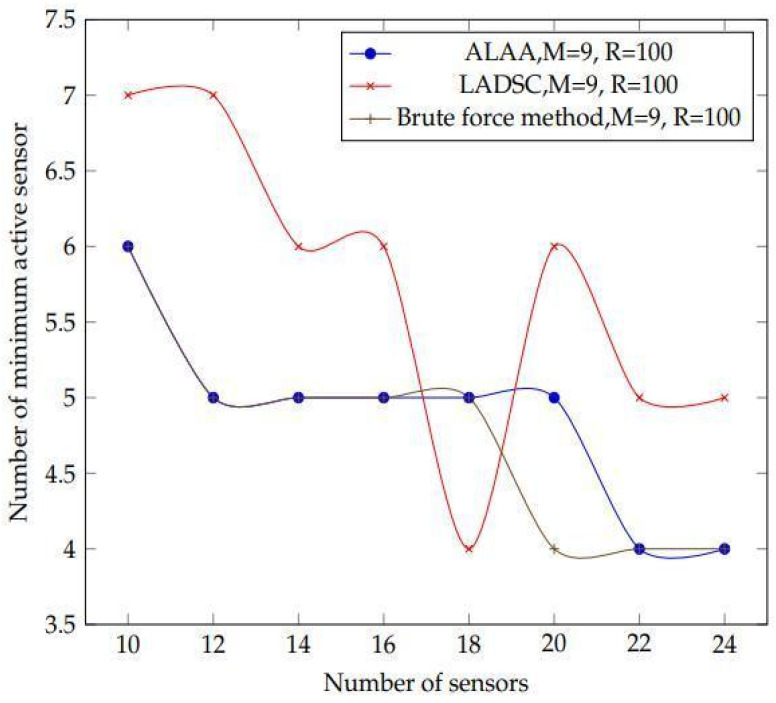
The plot shows results acquired by comparing the proposed adaptive learning automata algorithm (ALAA) to the learning automata disjoint coverage set (LADSC) algorithm and using the brute force approach to validate the results. There are nine targets in this experiment, with the sensor ranging from 9 to 25 and a sensing range of 100 m.

The outcomes of this experiment are shown in Figure 15. Green towers represent sensors, whereas red towers represent targets in the diagram. The number denotes the sensors that are covering the targets, and black circles represent their coverage. As a result of this outcome, it can be concluded that brute force is more effective than the suggested learning automata method in finding the best solution.

**Figure 16:**
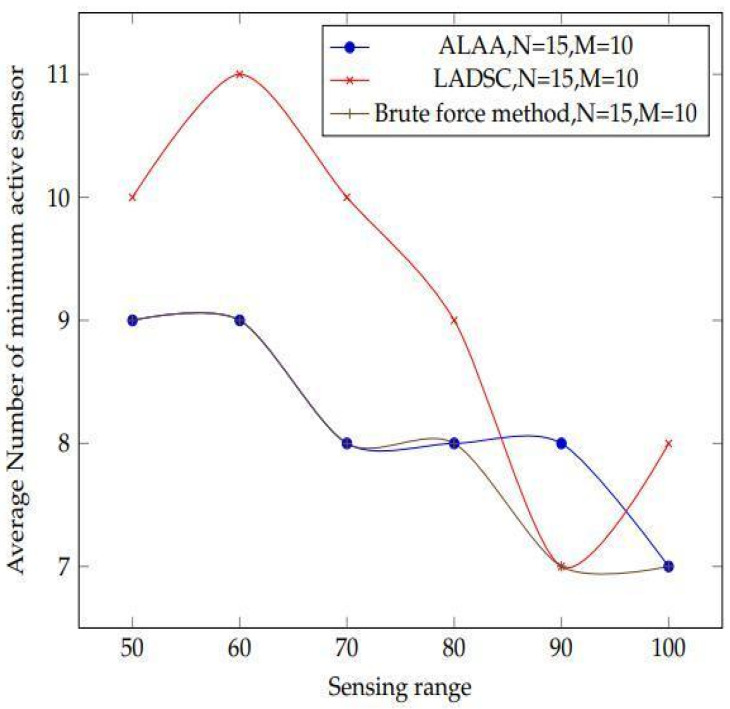
The plot shows results acquired by comparing the proposed adaptive learning automata algorithm (ALAA) to the learning automata disjoint coverage set (LADSC) Algorithm and using the brute force approach to validate the results. There are 15 sensors and 10 targets in this experiment, with sensing ranges ranging from 45 to 110 m.

This experiment compares the suggested learning algorithm against different algo- rithms in order to determine its efficiency (Figure 16). To compare the outcomes of the proposed learning automata method, the LADSC scheduling technique from [69,70,71] is used. LADSC outperforms other scheduling algorithms like MC-MIP, and Slijepcevic and Potkonjak in the experiments. Thus, we have decided to choose LADSC to benchmark against our approach [72].

**Figure 17:**
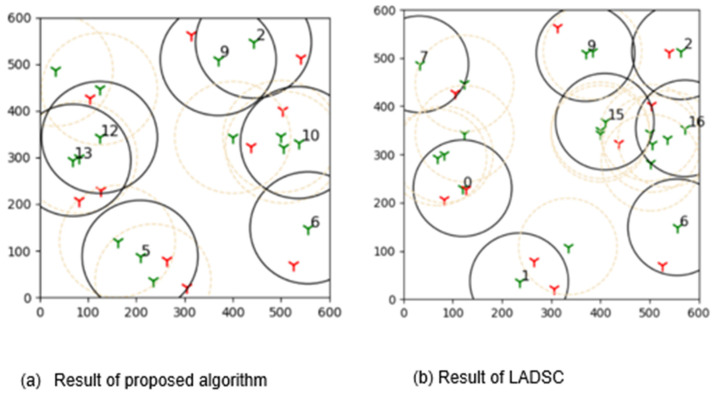
With a changing sensing range of sensors from 50 m to 100 m, simulation result achieved after design of the experiment utilising the proposed learning automata algorithm and LADSC algorithm.

The plot of the results obtained after constructing two algorithms is shown in Figure 17. The varied patterns of the findings are clearly seen in this diagram. In comparison to these two algorithms, the suggested method produces superior results. The LADSC algorithm demonstrates that they employ fewer active sensors at various points in the graph. However, they are not covering all of the targets in this scenario. Our suggested method, on the other hand, covers all of the objectives. As a result, the suggested method proves to be more consistent than the LADSC algorithm.

Figure 17 illustrates that when using the LADSC method, more sensors are required to cover all of the targets, whereas the suggested approach uses fewer active sensors. As a result of this finding, the suggested algorithm is capable of producing optimal outcomes.

## 5. Conclusions

This article focuses on resolving the wireless sensor network’s energy issue, as well as the target coverage problem. This necessitates the creation of an architecture and a machine learning algorithm. The adaptive learning automata algorithm is the name of this algorithm. The findings acquired after formulating the experiments using this approach were examined to see if they were valid in meeting the project’s objectives. It has also been compared to other algorithms mentioned in the project’s related work section to determine its efficiency. This method uses a learning approach to arrange sensors in order to conserve energy, thus, that they may choose whether to be active or sleep on their own. In the active mode, sensors are expected to use some energy to cover the accessible targets. To test the performance of the developed algorithm and architecture, several experiments and simulations are carried out. The suggested algorithm lays forth a way for determining the smallest number of active sensors required to cover all targets. In other words, by using the smallest number of active sensors, the network consumes the least amount of energy, and therefore this idea aids in energy conservation in wireless sensor networks. As a result, this research focuses on the idea of an energy-efficient target coverage network. Both small and big wireless sensor networks can benefit from this method. However, in order to obtain exact findings, the learning parameter value should be addressed throughout the formulation of experiments in this sort of network. The sensor’s sensing range has the same effect on both sorts of networks. Furthermore, while comparing related work algorithms in terms of sensing range and number of sensors, the findings demonstrate that our suggested method outperforms the comparison algorithm. Finally, all of the experimental data collected thus far demonstrate that the suggested method has justified the problem description and achieved the project’s aim. So, while constructing an actual sensor network, our suggested algorithm may be utilised as a useful technique for scheduling sensor nodes.

## Data Availability

Not applicable.
